# Hypertensive load predicts recovery of renal function for patients undergoing revascularisation for renal artery stenosis

**DOI:** 10.1038/s41598-025-86663-y

**Published:** 2025-01-28

**Authors:** Ben Edgar, Rob Pearson, Andrew Jackson, Callum Stove, Ram Kasthuri, Keith Hussey, Christian Delles, Colin Geddes, Patrick Mark, Giles Roditi, Linsay McCallum, David B. Kingsmore

**Affiliations:** 1 School of Cardiovascular and Metabolic Health, University of Glasgow, Glasgow, UK; 2 Glasgow Renal and Transplant Unit, Queen Elizabeth University Hospital, Glasgow, UK; 3 Department of Interventional Radiology, Queen Elizabeth University Hospital, Glasgow, UK; 4 Department of Vascular Surgery, Queen Elizabeth University Hospital, Glasgow, UK

**Keywords:** Blood pressure, Hypertension, Ischaemia, Renal artery stenosis, Renin-angiotensin system, Outcomes research, Renovascular hypertension, Reconstruction, Acute kidney injury, Chronic kidney disease, Renal artery stenosis

## Abstract

Renal ischaemia due to renal artery stenosis produces two differing responses - a juxtaglomerular hypertensive response and cortical renal dysfunction. The reversibility of renal impairment is not predictable, and thus renal revascularisation is controversial. This study aims to test the hypothesis that the hypertensive response to renal ischaemia reflects viable renal parenchyma, and thus could be used to predict the recovery in renal function. A retrospective analysis was performed of all patients who had renal revascularisation for renal impairment in a defined geographical area (West of Scotland, population 2.4 million) between 2008 and 2024. Clinical records were used to determine the pre-intervention blood pressure, anti-hypertensive medication load and renal function, and post-intervention outcomes. The Hypertensive Index (HTi), a combined measure of systolic blood pressure and antihypertensive drug load, was used as a measure of pre-intervention hypertensive response. 75 patients had intervention for renal impairment over 15 years (68 endovascular, 7 open). Mean pre-intervention serum creatinine of 323 µmol/L was reduced to 191 umol/L at discharge and 182 µmol/L at 6-month follow-up. Refractory hypertension (HTi > 120) was associated with a significant benefit from revascularisation with improved renal function (*p* = 0.003) and reduced risk of future dialysis (*p* = 0.001). Renal impairment with no hypertensive response was highly predictive of the need for future dialysis. The hypertensive index is a good predictor of the impact of renal revascularisation on improving renal function with good outcomes in selected patients, and the absence of this is an indicator of chronic non-reversible renal dysfunction.

## Introduction

Radiological renal artery stenosis (RAS) is relatively common, being found in up to 68% of those with heart failure and chronic kidney disease^1^. However, it is difficult to be certain of the extent that RAS seen on imaging contributes to presenting cardiovascular or renal symptoms. This may be due to the variation in type and severity of symptoms that may arise from RAS that includes acute cardio-renal syndromes to chronic conditions such as hypertension (from renin-angiotensin-aldosterone system and sympathetic nervous system activation), impairment of renal function (from acute kidney injury to fibrosis and ischaemic atrophy) and incidental discovery on imaging for apparently unrelated clinical symptoms.

Despite a clear association between an aetiological cause (stenosis of a renal artery reducing renal blood flow and causing critical renal ischaemia) and the varied pathophysiological outcomes, there remains much scepticism in the benefits of renal re-vascularisation. Partly this may relate to the inter-relationship of co-existent risk factors that can cause hypertension or renal impairment independently of RAS making it difficult to attribute cause and effect. More importantly, large randomised controlled trials (RCTs) failed to show a reno-protective benefit with the addition of renal artery stenting to best medical therapy^2,3^. However, these RCT have several critical limitations, perhaps the most important of which is that patients were analysed on the basis of an anatomical lesion rather than the symptoms and indication for treatment. The importance of this was shown in subsequent less well publicized re-analyses that showed potential benefits from stenting in selected sub-groups of symptomatic patients^4,5^.

The role of revascularisation of RAS to improve renal function is particularly controversial. It is estimated that 15% of patients with end-stage renal failure (ESRF) have RAS^6–8^, and several smaller cohort series have reported a benefit from RAS stenting in mild or moderate CKD^9–13^. Sub-studies of RCT of blood pressure management have indicated that tighter target blood pressure parameters were actually associated with an increased incidence of renal impairment (ACCORD and SPRINT secondary analysis)^14^.

This uncertainty may partly reflect the complex physiology of renal blood flow. Renal tubular and cortical function is reliant on very high renal blood flow whereas only a small fraction of the delivered oxygen is required for viability under normal physiological conditions^15,16^. Reductions in renal blood flow therefore may cause impaired renal function, but still permit sufficient oxygen for cell viability and stimulation of the juxta-glomerular hypertensive response.

Previously we have demonstrated a direct relationship between the severity of pre-intervention hypertensive load (the hypertensive index, HTi – a combination of antihypertensive drug load and blood pressure excess) and the improvement in blood pressure control following renal artery stenting^17^. This confirmed that the hypertensive drive from renal ischaemia (the HTi) was a reflection of critically ischaemic but viable juxta-glomerular cells producing an active renin-angiotensin response.

The aim of this study was therefore to determine if the pre-intervention hypertensive load, i.e. the hypertensive index (HTi), could also predict a beneficial impact on renal function following renal revascularisation performed for impaired renal function.

## Materials and methods

A retrospective analysis was performed of all patients who had renal impairment at the time of undergoing renal revascularisation in a defined geographical area (the West of Scotland, population 2.4 million) between the years 2008 and 2024. Patients were identified through a word-search of the radiology information system in which all imaging and interventions are reported, and from a prospectively maintained database. All patients with renal impairment at the time of renal revascularisation were included in the analysis, with patients excluded in whom the sole aim was to improve the control of refractory hypertension in the context of mild CKD 3.

Renal impairment was defined as a eGFR < 60 ml/min/1.73m^2^according to the CKD-EPI Creatinine Eq. (2021)^18^. Acute renal dysfunction was defined as a rise in serum creatinine concentration by 25% of its baseline figure within the 6 months preceding intervention. Chronic renal dysfunction was defined by stable sub-normal function that had not deteriorated by 25% in the preceding 6 months. Renal function is described in terms of serum creatinine concentrations rather than eGFR due to the dynamic nature of rapidly changing creatinine levels in the time period surrounding intervention, which would render eGFR measurements unreliable. To compensate for this, patients were stratified into groups with baseline eGFR greater than or less 30 ml/min/1.73m^2^ for the purposes of statistical analysis. The results were the same if eGFR was used.

The Hypertensive Index^17^ (HTi) was the value calculated as below:

### **HTi = [the mean systolic blood pressure – 120] X [the number of antihypertensive medications + 1]**

The blood pressure measurements were determined by taking the mean value of 3 readings using an automated oscillometric device under office conditions, and the number of antihypertensive medications taken from the electronic patient record. A critical HTi was based on the American Heart Association definition of refractory hypertension as ‘blood pressure which remains elevated (≥ 140mmHg SBP) despite the use of ≥ 5 antihypertensive agents’ which equates to a hypertensive index of 120 or above^19^.

Proteinuria was adapted into a binary variable based on the KDIGO classification of ‘Normal to Mildly Increased (A1)’, ‘Moderately Increased (A2)’ or ‘Severely Increased (A3)’, with those falling into the A1 class considered as not having significant proteinuria^20^.

Procedural details and outcomes (including blood pressure, medication, and renal function) were recorded. The outcomes of intervention were described from the time of intervention. In those who had commenced haemodialysis prior to intervention, pre-intervention serum creatinine concentration was recorded as the level prior to commencing dialysis. Post-intervention data relates to the time of discharge from the hospital admission or 4 weeks post-procedure if not discharged. Follow up data relates to 6 months post-procedure. Patients requiring pre-intervention haemodialysis who did not achieve recovery of renal function were assumed to have no change in serum creatinine concentration.

There were 4 peri-procedural deaths: one at day 1 following a successful renal artery stent for acute occlusion, and one patient sustained an endovascular injury to a replaced right hepatic artery which was cannulated as a consequence of aberrant anatomy and died on the 3rd post-procedure day. Renal artery dissection occurred twice – both patients initially recovered but died as a result of comorbid illness (seizures; myocardial infarction) at 16 and 22 days respectively. These patients were excluded from the analysis due to the absence of follow up data.

#### Statistical analysis

Data was collated using Microsoft Excel (Version 16.53 © Microsoft 2021). Statistical analysis was performed using RStudio (Version 1.4.1717 © 2009–2021 RStudio, PBC). Data distribution was assessed using the Shapiro-Wilk test. Means were compared by Wilcoxon rank-sum test or Kruskal-Wallis rank sum test as appropriate. Univariate analysis included any independent variable suspected by the authors to potentially influence outcomes. Variables which showed statistical significance in univariate analysis were further assessed in a multivariate model. The impact of revascularisation on renal function was assessed in a logistic regression model with a ≥ 25% reduction in serum creatinine as the dependent variable. Time-to-event analysis was performed using a Cox proportional hazards regression model, with patients censored at the time of death or the end of the follow up period.

## Ethics

All participants provided written informed consent, and all study procedures were in concordance with the Declaration of Helsinki, 1975 (revised 2000). Formal ethical approval was not required as per national legislation produced by the Health Research Authority^21^.

## Results

Over a 15-year time-period, 75 patients had revascularization with the aim of improving renal function. The mean age was 66, 47% were male, and other comorbidities included diabetes (20%), coronary heart disease (49%), cerebrovascular disease (27%) (Table 1). Before intervention, the mean serum creatinine concentration was 323 (+/- 286) µmol/L, 64% had proteinuria and renal dysfunction was classified as acute in 47%. The mean hypertensive index (HTi) was 307 (SD 176, range 30–756). Intervention was by endovascular renal artery stent in 68 (90%), open ilio-renal bypass in 5 (7%) and autotransplantation in 2 (3%).

Renal artery stenosis was bilateral in 65% (*n* = 49), with 39% (*n* = 29) considered to have a single functioning kidney due to atrophy of one kidney (average of 3.2 cm difference in craniocaudal length between “functioning” and “non-functioning” kidney). There was unilateral renal agenesis in 5 patients (7%), and previous unilateral nephrectomy in 2 (3%). Intervention was unilateral in 86% (*n* = 65), with the median size of the kidney being re-vascularised being 10.5 cm in cranio-caudal length (IQR 9.1–11.9 cm).

**Table 1:** Study cohort demographics.

	*N* = 75
Age	
Mean (SD, Range)	66 (10, 34–83)
Gender, n (%)	
Female	40 (53%)
Male	35 (47%)
Diabetes mellitus, n (%)	15 (20%)
Ischaemic Heart Disease, n (%)	37 (49%)
Cerebrovascular Disease, n (%)	20 (27%)
Aetiology, n (%)	
ARAS	68 (91%)
Acute Aortic Syndrome	6 (8.0%)
Type B Aortic Dissection	1 (1.3%)
Pre-Intervention Serum Creatinine^1^	
Mean (SD, Range)	323 (286, 89 − 2,041)
Pre-Intervention eGFR^2^	
Mean (SD, Range)	24 (15, 3–59)
Pre-Intervention HTi^3^	
Mean (SD, Range)	307 (177, 30–756)
Proteinuria, n (%)^4^	
Normal to Mild	27 (36%)
Moderate	24 (32%)
Severe	24 (32%)
Renal Dysfunction, n (%)	
Acute	35 (47%)
Gradual	40 (53%)
Required RRT, n (%)	20 (27%)
Revascularisation, n (%)	
Endovascular	68 (91%)
Open	7 (9.3%)

^1^ umol/L; ^2^ ml/min/1.73 m2; ^3^ HTi = Hypertensive Index; ^4^ KDIGO Classification.

**Fig. 1:**
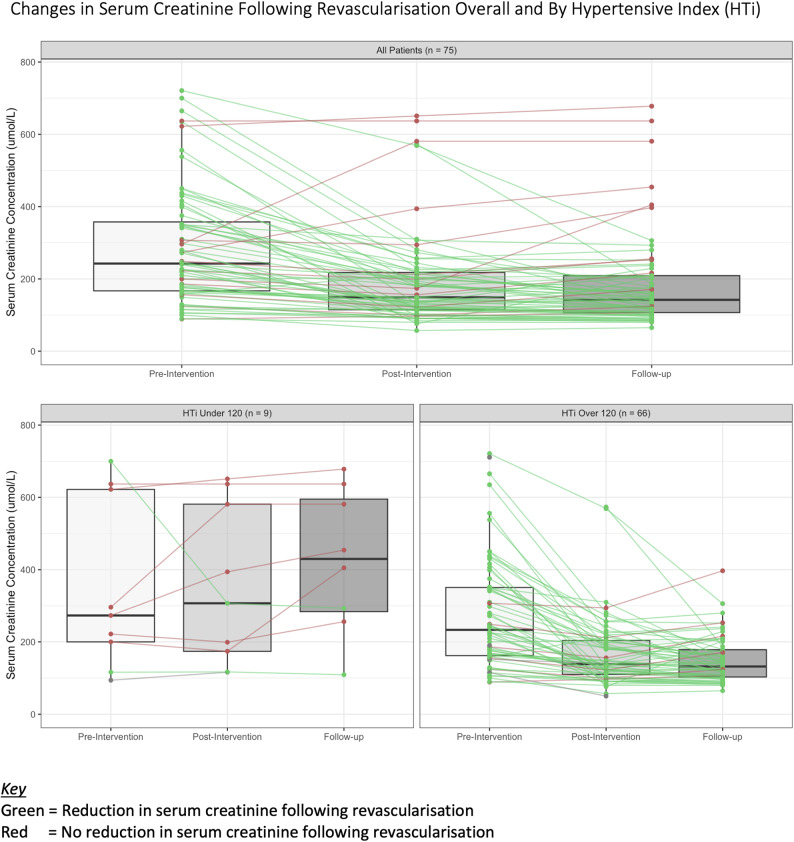
Combined boxplot and line graph demonstrating reductions in serum creatinine following revascularisation in the whol study cohort and stratified by the pre-intervention hypertensive index.

Overall, renal function significantly improved by 30% following intervention and was maintained at 6-months (SCr: 323 pre-intervention: 191 µmol/L on discharge; 182 µmol/L at six months, Fig. 1). Systolic blood pressure was also reduced by an average of 22%, (from 185mmHg to 145mmHg on discharge and 147mmHg at six months), with a requirement for one less antihypertensive medication to maintain this blood pressure (mean 4 antihypertensives pre-intervention, 2 at discharge and 3 at follow up). The HTi was 72% better following intervention (mean: 307 pre-intervention, 84 at discharge, 104 at follow up).

## Predictive measures of outcome

**Fig. 2:**
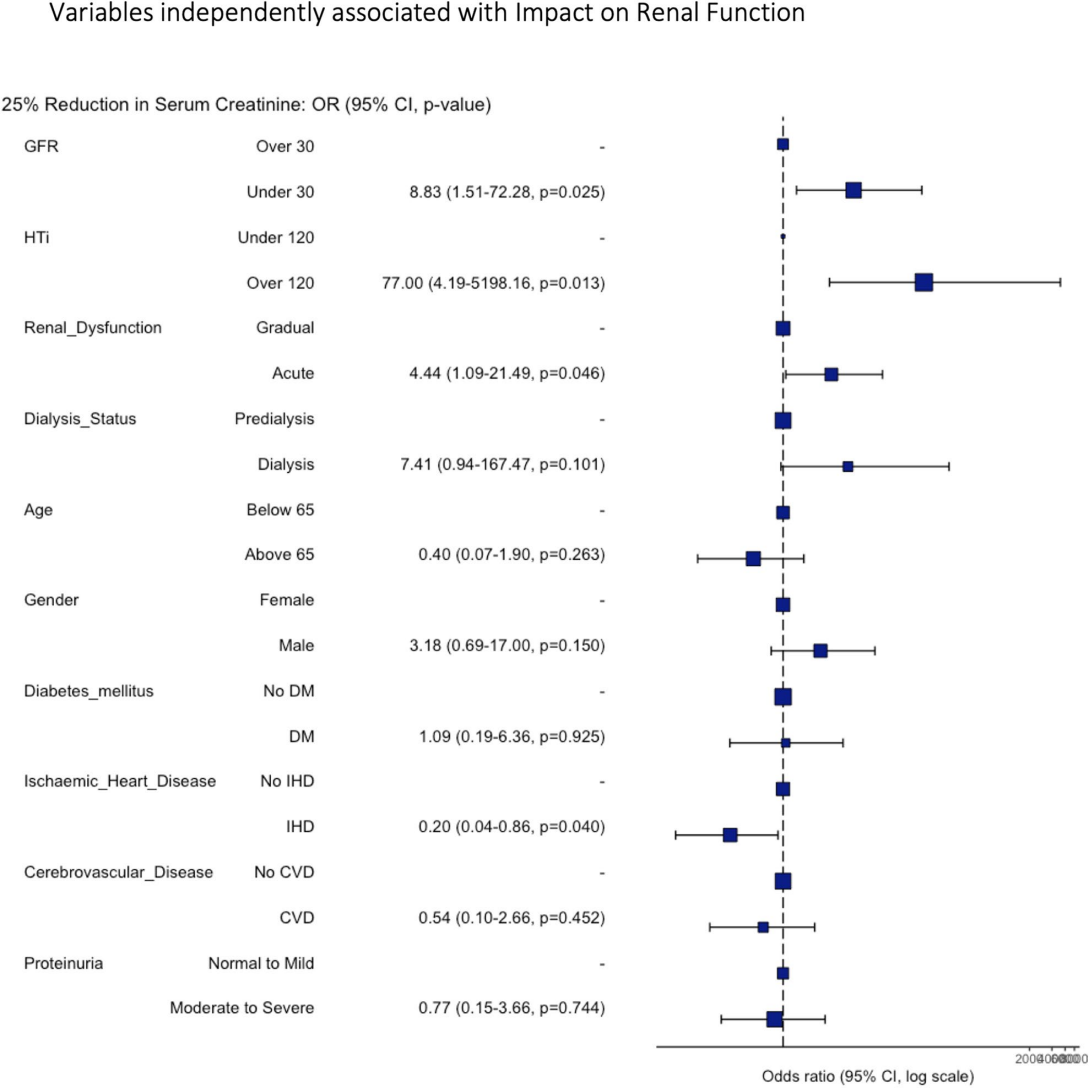
Forest plot demonstrating variables which are independently associated with the impact of revascularisation on renal function.

On univariate analysis, most clinical features did not correlate with the response to revascularisation (age (*p* = 0.3, X^2^ 1.08), sex (*p* = 0.6, X^2^ 0.21), aetiology of renal dysfunction (*p* = 0.072, X^2^ 1.6), method of revascularisation (*p* = 0.7, X^2^ 0.06), diabetes (*p* = 0.7, X^2^ 0.07), ischaemic heart disease (*p* = 0.08, X^2^ 2.9), cerebrovascular disease (*p* = 0.4, X^2^ 06), and proteinuria (*p* = 0.4, X^2^ 1.8)). Three factors were significantly associated with an improvement in renal function: progression of renal dysfunction (acute vs. chronic, X^2^ 7.8), eGFR (< 30 vs. 30+, X^2^ 4.8) and HTi (< 120 vs. > 120, X^2^ 6.3) (Fig. 1; Table 2). These characteristics remained independently significant on multivariate analysis (Fig. 2; Table 3). Interestingly, no clinical characteristics of patients discriminated between those with a high HTi vs. low HTi (age *p* = 0.5, X^2^ 0.4; sex *p* = 0.8, X^2^ 0.04; diabetes *p* = 1, X^2^ < 0.05; ischaemic heart disease *p* = 0.14, X^2^ 2.14; cerebrovascular disease *p* = 0.9, X^2^ 0.006; aetiology *p* = 0.8, X^2^ 0.26; rate of renal dysfunction *p* = 0.8, X^2^ 0.04; type of revascularisation *p* = 0.4, X^2^ 0.6; and proteinuria *p* = 0.7, X^2^ 0.8).

**Table 2:** Renal function response by patient characteristics.

	Pre-Intervention	Post-Intervention			Follow-up		
	µmol/L^1^	Creatinine Change		*p*-value^2^	Creatinine Change		*p*-value^2^
		µmol/L^1^	%		µmol/L^1^	%^1^	
Pre-Intervention GFR							
Over 30	137 (34)	−23.4 (30)	−16%	**< 0.001**	−27 (27)	−19%	**< 0.001**
Under 30	406 (310)	−181 (274)	−36%		−202 (324)	−37%	
Pre-Intervention HTi							
Over 120	320 (294)	−148 (240)	−35%	**0.003**	−173 (280)	−39%	**0.003**
Under 120	351 (236)	+ 2 (190)	+ 11%		+ 50 (227)	+ 32%	
Rate of Renal Dysfunction							
Acute	417 (375)	−199 (325)	−37%	**0.0016**	−236 (375)	−44% (36)	**0.005**
Gradual	242 (132)	−73 (99)	−23%		−74 (128)	−21%	

^**1**^Mean (SD).

^**2**^Wilcoxon rank sum exact test; Wilcoxon rank sum test; Kruskal-Wallis rank sum test.

**Table 3:** Multivariate Analysis of Factors Associated with improvement in renal function.

Impact on Renal Function (25% Reduction in Serum Creatinine)		No	Yes	OR (95% CI, *p*-value) (univariable)	OR (95% CI, *p*-value) (multivariable)
eGFR	Over 30	14 (60.9)	9 (39.1)	-	-
	Under 30	15 (30.6)	34 (69.4)	**3.53** **(1.28–10.25**,**p = 0.017)**	**8.83** **(1.51–72.28**,**p = 0.025)**
HTi	Under 120	7 (87.5)	1 (12.5)	-	-
	Over 120	22 (34.4)	42 (65.6)	**13.36** **(2.19–258.23**,**p = 0.019)**	**77.00** **(4.19–5198.16**,**p = 0.013)**
Renal Dysfunction	Gradual	22 (56.4)	17 (43.6)	-	-
	Acute	7 (21.2)	26 (78.8)	**4.81** **(1.75–14.49**,**p = 0.003)**	**4.44** **(1.09–21.49**,**p = 0.046)**
Dialysis	Pre-Dialysis	25 (47.2)	28 (52.8)	**-**	**-**
	Dialysis	4 (21.1)	15 (78.9)	**3.35** **(1.05–12.96**,**p = 0.054)**	**-**
Age	Below 65	9 (29.0)	22 (71.0)	**-**	**-**
	Above 65	20 (48.8)	21 (51.2)	**0.43** **(0.15–1.13**,**p = 0.094)**	**-**
Gender	Female	16 (43.2)	21 (56.8)	**-**	**-**
	Male	13 (37.1)	22 (62.9)	**1.29** **(0.50–3.35**,**p = 0.598)**	**-**
Diabetes	No DM	22 (38.6)	35 (61.4)	**-**	**-**
	Yes	7 (46.7)	8 (53.3)	**0.72** **(0.23–2.31**,**p = 0.572)**	**-**
IHD	No IHD	10 (28.6)	25 (71.4)	**-**	**-**
	Yes	19 (51.4)	18 (48.6)	**0.38** **(0.14–0.99**,**p = 0.051)**	**-**
CVD	No	19 (36.5)	33 (63.5)	**-**	**-**
	Yes	10 (50.0)	10 (50.0)	**0.58** **(0.20–1.64**,**p = 0.299)**	**-**
Proteinuria	Normal to Mild	10 (40.0)	15 (60.0)	**-**	**-**
	Moderate to Severe	19 (40.4)	28 (59.6)	**0.98** **(0.36–2.63**,**p = 0.972)**	**-**

## Impact on future dialysis requirement and survival

**Fig. 3:**
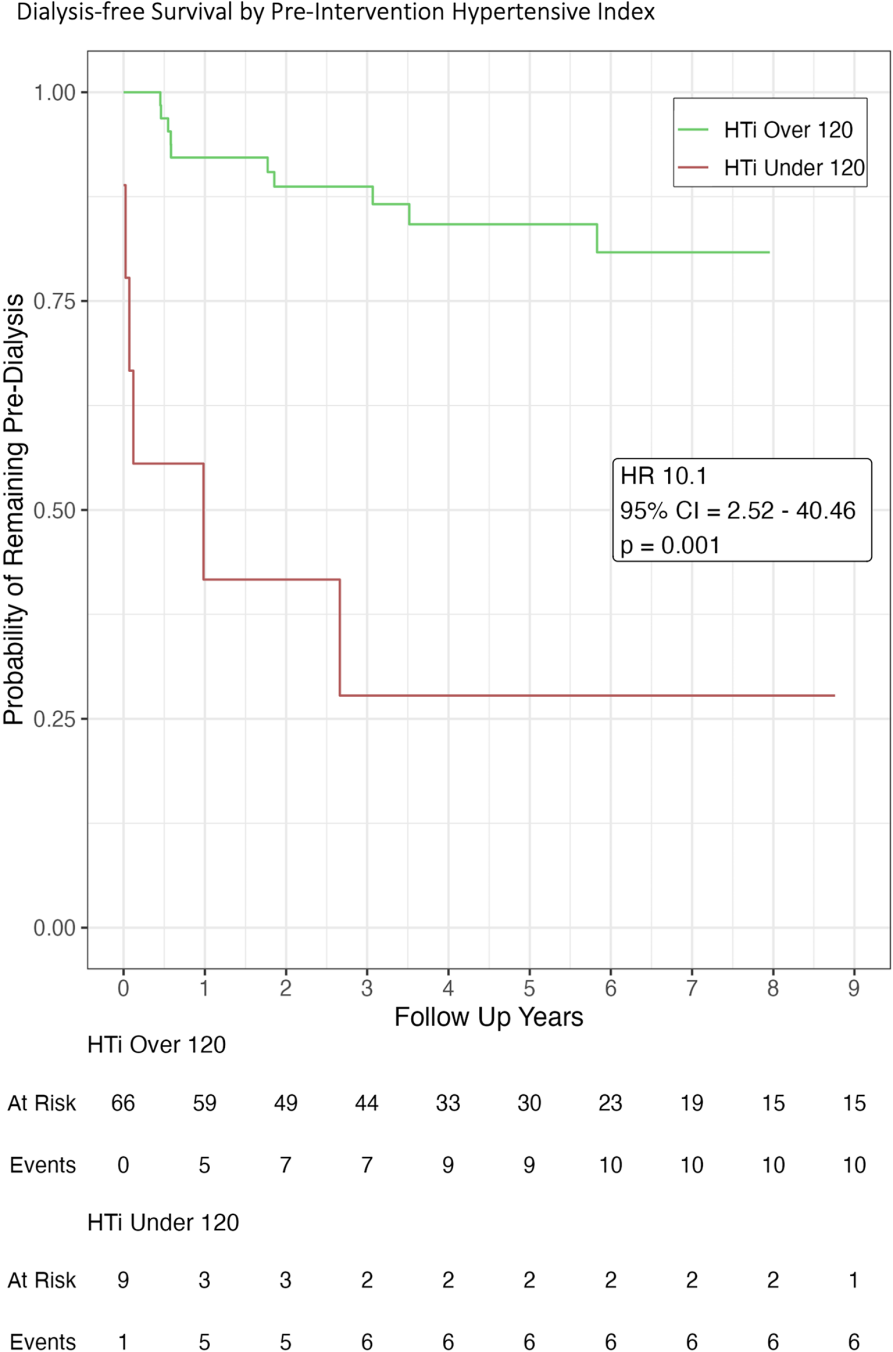
Kaplan-Meier plot showing significant difference in dialysis-free survival based on the pre-intervention hypertensive index.

**Fig. 4:**
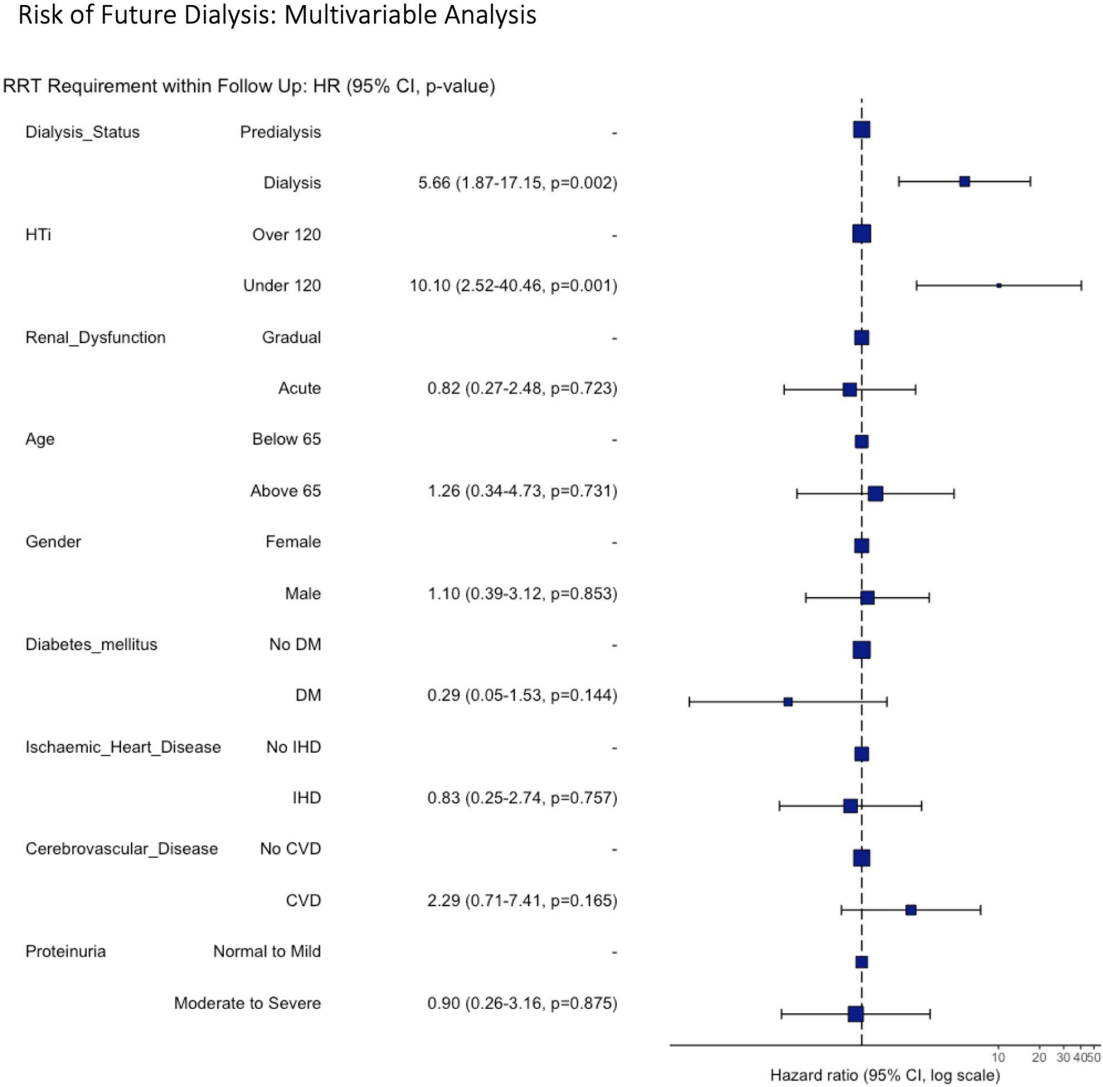
Forest plot demonstrating the association of patient variables the risk of future dialysis requirement following revascularisation.

Multivariate analysis showed the pre-intervention hypertensive load to be an effective predictor of the risk of future dialysis: those with a HTI below 120 were approximately 10 times more likely to require dialysis within the follow up period (HR 10.1, 95% CI 2.52–40.46, *p* = 0.001, Fig. 3). Those who required dialysis prior to revascularisation were also more likely to require dialysis within the follow up period (HR 5.66, 95% CI 1.87–17.15, *p* = 0.002, Fig. 4).

**Fig. 5:**
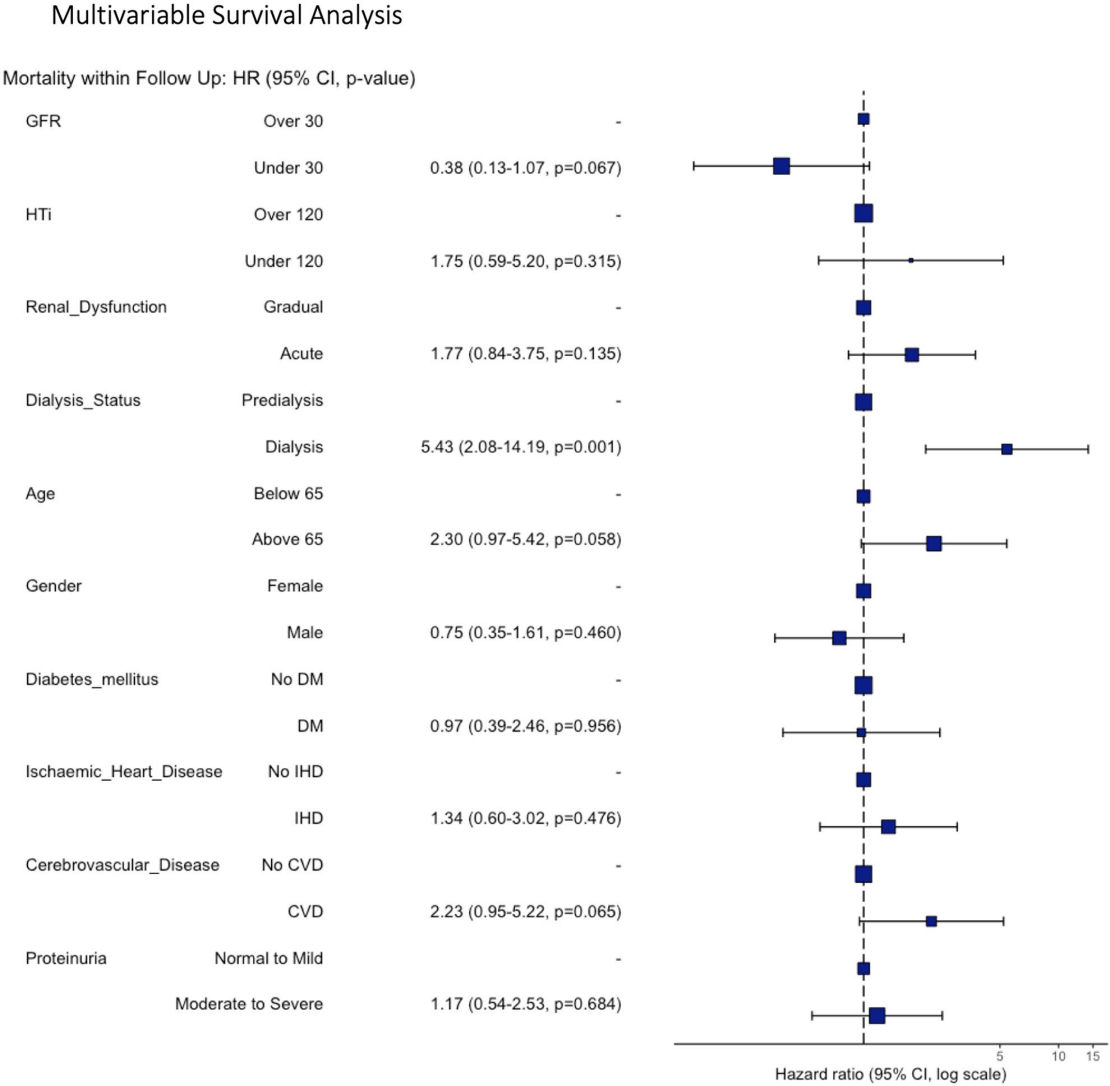
Forest plot demonstrating the association of patient variables the risk of mortality within the follow up period.

50% of patients were alive at the end of the follow-up period (total 174,046 follow up days). Pre-intervention dialysis was an independent predictor of overall survival, increasing the likelihood of all-cause mortality (HR 5.43, 95% CI 2.08–14.19, *p* = 0.001, Fig. 5). Mortality during follow up was increased in those without refractory hypertension (HTi < 120) however this did not meet statistical significance in multivariable analysis when adjusted for confounding variables (HR 1.75, 95% CI 0.59–5.20, *p* = 0.315).

## Discussion

Although it is recognised that renal function can potentially recover from an acute ischaemic insult, it is difficult to predict the impact on renal function following revascularisation for an individual patient. Thus, the aim of this analysis was to determine if the hypertensive load (a presumed marker of juxtaglomerular apparatus viability) is indicative of the potential for recovery of renal function. This analysis has shown that of three clinical factors that are independently associated with a significant improvement in renal function following revascularisation, the most important is the HTi. Interestingly, no other clinical factors including diabetes, previous cardiac diseases or even proteinuria correlate with the HTi. This supports the theory that the juxta-glomerular hormonal response to reduced renal perfusion is a good indicator of glomerular-tubular viability that can recover function following reperfusion. Importantly, the beneficial results were seen even when the GFR was low. This situation is analogous to critical ischaemia in the peripheral circulation or mesenteric beds in which the indication for intervention is the presence of critical symptoms such as rest pain or mesenteric angina. In the renal tissues, the hypertensive response (antihypertensive drug requirement and high blood pressure) may be seen as analogous to the rest pain seen in critical limb ischaemia.

These findings seem contrary to other RCT in which renal artery stenting did not lead to any benefit in renal function. However, it is likely that this reflects case-selection in the failed RCT and this report: CORAL and ASTRAL were inclusive and performed in a very heterogenous group of patients that had widely varying severity of stenosis and variable physiological impact. Both trials have been subject to criticism of their methodology: for the ASTRAL trial the inclusion criteria were a ‘substantial stenosis > 40%, physician equipoise on the benefits of stenting, or unexplained renal dysfunction’. Thus, many patients were included in whom a benefit was unlikely, and patients were purposefully excluded if intervention was felt likely to be beneficial. The CORAL trial had several critical limitations largely related to poor recruitment (leading to revision of the inclusion criteria and endpoints after recruitment had commenced), allowing the inclusion of patients who had minimal symptoms (29% had a Bp < 140 mmHg), and participation (21% lost to follow-up and crossover). Perhaps most importantly, patients were analysed on the basis of an anatomical lesion rather than the symptoms and indication for treatment (hypertension, acute or chronic renal dysfunction, or cardio-renal syndromes). This seems supported by sub-group analyses of only patients with severe stenosis and symptoms in whom a benefit was found^22^. Other sub-group analyses of RCT reported that tighter blood pressure control was associated with an increase in incident renal disease. However, the aetiology of subsequent renal impairment was uncertain, and potentially could even relate to underlying renal artery stenosis given the prevalence of significant renal artery stenosis in this population^14^.

Whilst the concept that the HTi would allow discrimination of patients who may benefit from renal revascularisation is very encouraging, there are many limitations to this work. Importantly, this is a small series of a very selected cohort with an unknown number of patients who either were not referred for consideration or turned down for an intervention. The small numbers of patients with a low HTi in the analysis would reduce the negative predictive value for the HTi. The data presented here is essentially the opposite of the larger RCTs with broad, inclusive selection criteria that dilute the benefit by including widely differing patient groups: we present the outcomes for a single technical approach, for a single indication, using an endpoint that is related to the indication for the procedure.

A further potential limitation is the absence of functional imaging techniques to grade the haemodynamic effects of the stenotic lesions. However, whilst perfusion-weighted imaging can quantify the functional significance of a stenotic lesion^23^and delineate renovascular disease from renal parenchymal disease^24^, there is no evidence that it can predict the physiological response to revascularisation at a cellular level. Ultrasonographic measures of perfusion have supported the theory that revascularisation is less likely to be beneficial once glomerulosclerosis is developed^25^, and this is in keeping with the outcomes seen when comparing acute and chronic renal dysfunction in this study.

Serum creatinine concentration has been used as the surrogate marker of renal function. Creatinine levels may be influenced by age, sex, race and body habitus, and may be misleading when comparing heterogenous populations. However, in the short timeframe captured by this study, significant changes in individual body habitus are unlikely, and the main utility is in comparing the intra-individual variation in serum creatinine over the time of intervention. Accurate radio-isotopic measures of GFR are not readily available and estimated GFR levels are only reported up to a level of ‘greater than 60’, making statistical analysis problematic. This may obscure the full benefit obtained by some patients who returned to normal levels of renal function following revascularisation. Additionally, eGFR is not a reliable measurement for patients with rapidly changing creatinine levels, which is often the case in the immediate timeframe following revascularisation. Importantly, when the statistical analysis was performed with eGFR in lieu of creatinine, the outcomes were the same.

Examination of biochemical markers of hyperreninemic hyperaldosteronism such as serum renin concentrations and/or urinary potassium concentrations may have been a useful adjunct in confirming the diagnosis of renovascular hypertension. Unfortunately neither is routinely tested in local practice, as diagnosis relies largely on clinical correlation with radiological findings. This would be enlightening data to collect in any future prospective studies of renal revascularisation.

The definition of the HTi may also be criticised in being an overly broad approximation that does not discriminate between drug types, adherence, or dose. However, there is little literature to show significant differences in the efficacy between drug types, particularly in this patient cohort, and the number of medication types is an accurate reflection of disease resistance and drug burden^26^. Similarly, the variety of interventions performed may be criticised, but the fundamental point of this article is to demonstrate that re-vascularisation in the right patients is worthwhile – how this is performed relates to patient and service factors.

In our experience, a multi-disciplinary approach that can offer both open surgical and endovascular interventions allows optimisation of the treatment by individual risks and comorbidity. In our unit each case is reviewed and discussed at a bimonthly multidisciplinary meeting attended by vascular and renal transplant surgeons, interventional radiologists, and a range of physicians with special interests in nephrology and hypertension.

In summary, this small study shows that in a cohort of patients, selected by the symptoms of renal ischaemia in the context of renal impairment, there is a clear benefit in re-vascularisation which justifies the relatively small risk, and underlines the importance of proper patient selection. The HTi offers promise as a prognostic tool to aid decision making.

## Data Availability

The datasets generated during and/or analysed during the current study are not openly available for reasons of patient confidentiality however anonymised data may be available from the corresponding author on reasonable request.
